# Comparative Study on the Damage Characteristics of Asphalt Mixtures Reinforced with an Eco-Friendly Basalt Fiber under Freeze-thaw Cycles

**DOI:** 10.3390/ma11122488

**Published:** 2018-12-07

**Authors:** Yongchun Cheng, Wensheng Wang, Yafeng Gong, Shurong Wang, Shuting Yang, Xun Sun

**Affiliations:** College of Transportation, Jilin University, Changchun 130025, China; chengyc@jlu.edu.cn (Y.C.); wangws17@mails.jlu.edu.cn (W.W.); wangsr@jlu.edu.cn (S.W.); yangst18@mails.jlu.edu.cn (S.Y.); sunx18@mails.jlu.edu.cn (X.S.)

**Keywords:** asphalt mixture, basalt fiber, freeze-thaw cycle, damage characteristics

## Abstract

The main distresses of asphalt pavements in seasonal frozen regions are due to the effects of water action, freeze-thaw cycles, traffic, and so on. Fibers are usually used to reinforce asphalt mixtures, in order to improve its mechanical properties. Basalt fiber is an eco-friendly mineral fiber with high mechanical performance, low water absorption, and an appropriate temperature range. This paper aims to address the freeze-thaw damage characteristics of asphalt mixtures (AC-13) reinforced with eco-friendly basalt fiber, with a length of 6 mm. Based on the Marshall design method and ordinary pavement performances, including rutting resistance, anti-cracking, and moisture stability, the optimum asphalt and basalt fiber contents were determined. Test results indicated that the pavement performances of asphalt mixture exhibited a trend of first increasing and then deceasing, with the basalt fiber content. Subsequently, asphalt mixtures with a basalt fiber content of 0.4% were prepared for further freeze-thaw tests. Through the comparative analysis of air voids, splitting strength, and indirect tensile stiffness modulus, it could be found that the performances of asphalt mixtures gradually declined with freeze-thaw cycles and basalt fiber had positive effects on the freeze-thaw resistance. This paper can be used as a reference for further investigation on the freeze-thaw damage model of asphalt mixtures with basalt fiber.

## 1. Introduction

The asphalt pavement has been widely used in flexible pavement constructions, with a rapid growing trend [1,2]. Asphalt mixture is generally considered to be a complex porous material that includes bitumen, aggregates, fillers, as well as voids [3,4]. However, due to some environmental factors, there are many distresses in asphalt pavements, such as spalling, crumble, pavement pothole, etc., especially in the seasonal frozen regions [5]. Therefore, researchers have been trying to modify asphalt mixtures and explore its freeze-thaw damage.

Fibers additives, such as cellulose fiber, polyester fiber, mineral fiber, etc., have been added into bitumen and proved to be an effective reinforcement material for asphalt mixtures [6,7,8,9]. Basalt fiber, as a novel kind of eco-friendly mineral fiber, was produced from basalt rocks with high mechanical properties, low water absorption, and its by-product can be directly degraded in the environment, without any harm [10]. Wang et al. [11,12] added basalt fiber into asphalt materials and evaluated their fatigue resistance by using direct tension, as well as fatigue tests. By means of an X-ray computed tomography technology (i.e., CT technology) and finite-element method, basalt fiber can release stress concentrations in critical areas and reduce fatigue damages. Gu et al. [13] compared and discussed basalt fiber and commonly used fibers and found that basalt fiber has a superior reinforcement effect on the high-temperature anti-rutting ability of bitumen mastic. Qin et al. [14] tested the reinforcement effects of basalt fibers, with lengths of 3, 6, and 9 mm asphalt mastics, with respect to the lignin fiber and the polyester fiber. Through leakage, penetration, strip-tensile and DSR tests, basalt fiber, especially, with a length of 6 mm, has excellent comprehensive performances, due to a steady three dimensional (3D) networking structure in bitumen mastics. Zhang et al. [15] carried out repeated and multi-stress creep tests and used Abaqus for analyzing the high-temperature performance of asphalt mastics. Then Zhang et al. [16,17] conducted the numerical simulations in Abaqus for the compressive creep and bending creep tests, for the purpose of analyzing the distribution effect and reinforcement mechanism of basalt fiber. Wang et al. [18] explored the optimization design of styrene-butadiene-styrene (SBS)-modified asphalt mixtures, with basalt fiber, with the assistance of a central composite design method. Test results indicated that asphalt mixtures, with basalt fiber, of 0.34% and a length of 6 mm, exhibited superior Marshall properties. Previous studies indicated that basalt fiber was effective in improving the mechanical properties of asphalt materials.

In recent years, experiments about the freeze-thaw cycle effects on asphalt mixtures, were also investigated by many researchers [19,20,21]. Xu et al. [22] employed the computed tomography (CT technology) to obtain and analyze internal images of asphalt mixtures, under different freeze-thaw cycles and investigated the influences of freeze-thaw cycles, on the evolution of internal air voids. Moreover, Xu et al. [23] studied the effects of freeze-thaw cycles on the thermodynamic characteristics of asphalt mixtures, based on the information entropy theory, CT, and digital image processing (DIP) technologies. The effects of freeze-thaw cycles on the permeability of asphalt mixtures have also been evaluated by means of a flow state, as well as water conductivity of asphalt mixtures [24]. Yan et al. [25] investigated the stone mastic asphalt (SMA) mixtures under the action of freeze-thaw cycles and evaluated the freeze-thaw resistance based on the Marshall design indicators and water stability. Badeli et al. [26] conducted the rapid freeze-thaw cycle test for asphalt mixture, using thermomechanical tests. Yi et al. [27] established the generalized Maxwell and Drucker-Prager model to evaluate the viscoelastic-plastic damage, under the condition of freeze-thaw cycles. Uniaxial compressive strength tests were carried out to investigate the mechanism of the freeze-thaw failure of asphalt mixtures. Nevertheless, efforts done for asphalt mixture with basalt fiber, under freeze-thaw cycles, are still limited in this area.

In this paper, asphalt mixtures (AC-13) reinforced with an eco-friendly basalt fiber with a length of 6 mm, were first designed by the Marshall design method, in order to determine the optimum asphalt content. Then optimum basalt fiber content could be also obtained, according to the ordinary pavement performances, such as rutting resistance, the indirect tensile stiffness modulus, and moisture stability. Subsequently, freeze-thaw cycle tests were performed for control and test groups of asphalt mixtures and the freeze-thaw damage characteristics were evaluated by a comparative analysis.

## 2. Materials and Methods

### 2.1. Raw Materials

**Figure 1:**
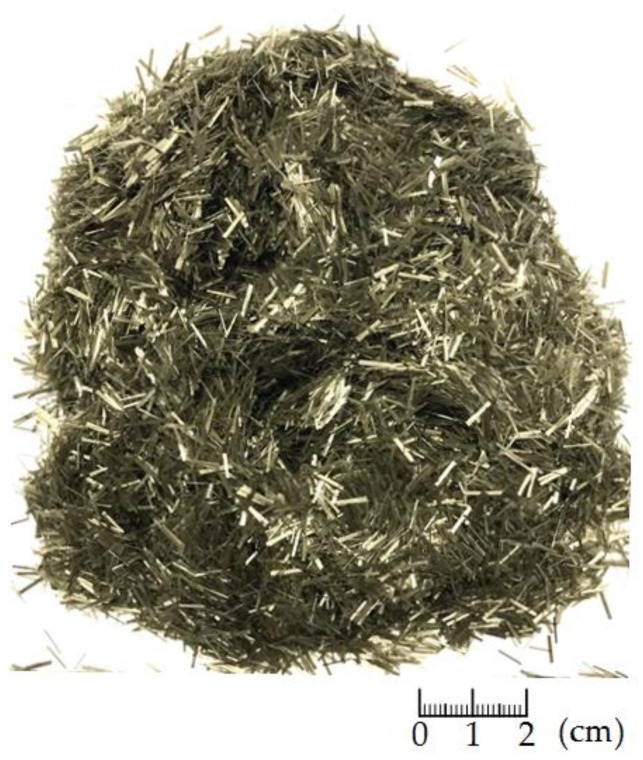
The golden-brown 6 mm long basalt fibers that were used in the study.

In this paper, bitumen of AH-90 was used, which was produced by the PetroChina Liaohe Petrochemical Company (Panjin, China). The basic physical performances of the AH-90 bitumen are presented in Table 1. Andesite mineral aggregates, which came from a local quarry in the Jilin Province, were chosen. Limestone powder was selected as the mineral filler for the bitumen mixture. The physical parameters of the aggregates and the filler are given in Table 2 and Table 3. Basalt fiber (shown in Figure 1) was obtained from the Jiuxin Basalt Industry Co., Ltd. (Changchun, China), the physical performances of which are listed in Table 4.

### 2.2. Sample Preparation

**Figure 2:**
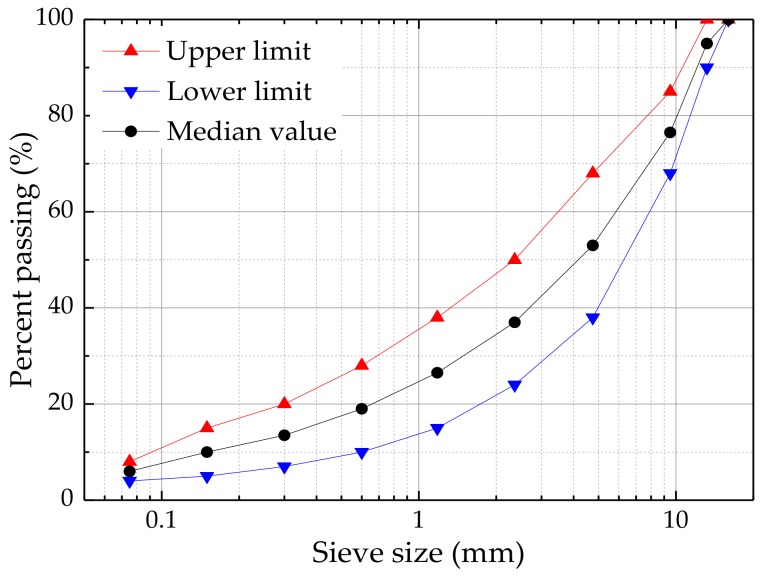
Gradation of the asphalt mixture (AC-13).

Traditional dense-graded asphalt mixture is a frequently-used asphalt mixture and is applied widely in the asphalt pavement construction in China [28]. The standard Marshall design method was adopted to prepare the asphalt mixture specimens [29]. Figure 2 presents the gradation curve of the asphalt mixture (AC-13) used in this study, the upper and lower limits, and selected median values of AC-13 are shown in Figure 2. In this paper, basalt fibers with a length of 6 mm was added into the asphalt mixtures, at four proportions of 0.2%, 0.3%, 0.4%, and 0.5% by a mass of asphalt mixture, respectively. According to the JTG E20-2011 [30], the detailed preparation procedures are presented as follows:

(i) The pre-heated aggregates mixed together with basalt fibers, in a mixing pot, for 90 s, in order to uniformly disperse the basalt fibers in aggregates.
(ii) The pre-heated bitumen AH-90 was weighted and poured into the mixing pot and the mixture was blended for 90 s.
(ii) The pre-weighted limestone powder was added into the mixing pot and then blended for 90 s.
(iv) Marshall specimens of AC-13, of a diameter 101.6 mm and a height of 63.5 mm, were prepared by compacting 75 blows on each side, and square slab specimens with dimensions of 300 mm × 300 mm × 50 mm, were prepared with the help of the wheel rolling [31].

### 2.3. Testing Procedure

**Figure 3:**
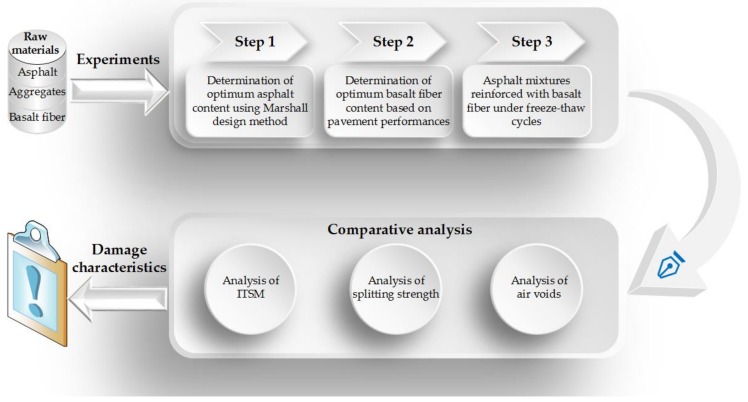
The research outline of this paper.

Figure 3 illustrates the research outline of this paper. First, raw materials of the asphalt mixtures were chosen, such as bitumen, aggregates, mineral powder, and the basalt fiber, listed in Section 2.1. Then, basalt fibers, of length 6 mm, were added into the asphalt mixtures, in different proportions of 0%, 0.2%, 0.3%, 0.4%, and 0.5% corresponding to the mass of the asphalt mixture, respectively. The optimum asphalt content for these asphalt mixtures could be determined by the Marshall design method described in Section 3.1. Subsequently, through ordinary pavement performances, including rutting resistance, anti-cracking, and moisture stability, the optimum basalt fiber content could be obtained (Section 3.2). Afterwards, the freeze-thaw cycle test was conducted for the asphalt mixtures, with the optimum basalt fiber content and without basalt fiber. Through the comparative analysis of air voids, splitting strength and indirect tensile stiffness modulus, the effects of the freeze-thaw cycles on the asphalt mixtures, could be addressed (Section 3.3).

### 2.4. Experimental Methods

#### 2.4.1. Marshall Design Method

Nowadays, the Marshall design method is used, extensively, in the asphalt pavement design and is also employed by the Chinese specification JTG E20-2011 [30]. The basic concepts of the Marshall mix-design method were originally developed by Bruce Marshall of the Mississippi Highway Department, around 1939, and then refined by the U.S. Army [32,33]. The Marshall design method seeks to select the optimum bitumen content at a desired density that satisfies the minimum stability and the range of flow values [29]. Compared to other design methods like the Superpave method, the Marshall design method is a proven method and requires relatively light, portable, and inexpensive equipment.

In this study, asphalt mixtures reinforced with basalt fiber were prepared on the basis of the Marshall design method and specimens were also tested, in order to obtain the Marshall design parameters, i.e., bulk density (*ρ_f_*), air voids (*VA*), voids in mineral aggregates (*VMA*), voids filled with asphalt (*VFA*), Marshall stability (*MS*), as well as the flow value (*FV*). In accordance with rule T0709 JTG E20-2011, bulk density (*ρ_f_*) can be determined through weighing the asphalt mixture specimens in air and water, following Equations (1) and (2). Afterwards, the *VA*, *VMA*, and *VFA* can be also calculated by using Equations (3)–(5).

*γ_f_* = *m_a_*/(*m_f_* − *m_w_*), (1)

*ρ_f_* = *γ_f_* × *ρ_w_*, (2)

*VA* = [1 − *γ_f_/γ_TMD_*] × 100, (3)

*VMA* = [1 − *γ_f_* × *Ps*/*γ_sb_*] × 100, (4)

*VFA* = [(*VMA* − *VA*)/*VMA*] × 100, (5)

where *ρ_w_* and *ρ_f_* are the density of water and bulk density of specimens; *m_a_*, *m_w_*, and *m_f_* represent the mass of the specimens in air, water, and the saturated surface dry mass, respectively; *γ_f_* is the bulk specific gravity; *γ_TMD_* is the theoretical maximum specific density which can be measured by vacuum sealing method; *P_s_* is the aggregate content percent by weight of mixture; *γ_sb_* is the bulk specific gravity of aggregates.

The Marshall test was performed to obtain the stability and flow values, according to rule T0709 in the JTG E20-2011 [30]. First, the prepared Marshall specimens (as described in Section 2.2) were conditioned in the water bath, at 60 °C, for half an hour. Afterwards, a force was applied on the side face, until the peak load. According to the indicator of the Marshall apparatus, the Marshall stability and flow could be obtained and recorded.

#### 2.4.2. High-Temperature Rutting Test

**Figure 4:**
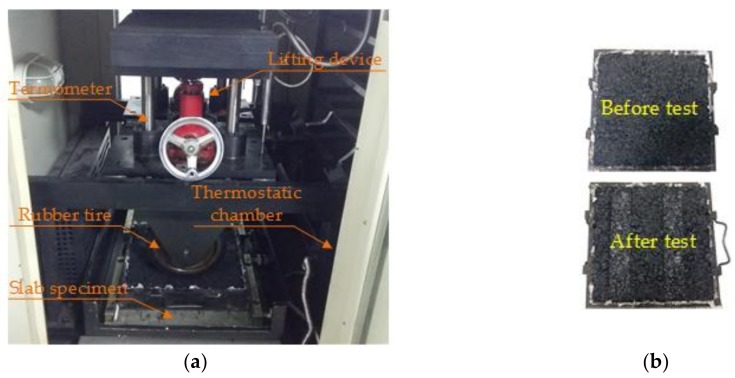
Rutting test in this paper: (**a**) Rutting test; and (**b**) slab specimens.

The rutting test is usually used for the high-temperature performance of asphalt mixtures and the test (shown in Figure 4a, China Highway Engineering Instrument Institute, Beijing, China) was carried out in accordance with rule T0719 in the JTG E20-2011 [30]. The detailed experimental procedures were as follows:

(i) Square slab specimens were placed in a dry environment of 60 ± 0.5 °C, for at least 5 h.
(ii) A rubber tire with a length of 50 mm was brought to the asphalt mixture slabs, for an hour, at a rolling speed of 42 ± 1 cycle/min, and the pressure of the loaded rubber tire was constant, i.e., 0.7 ± 0.05 MPa.
(iii) Then the rutting deflection could be measured vertically, per 20 s, by means of a linear variable differential transformer (LVDT).
(iv) The dynamic stability (*DS*) was defined by Equation 6 to quantitatively analyze the high-temperature rutting resistance. The rutting test was performed for three replicate specimens and the tested specimen is shown in Figure 4b.

*DS* = 15 × *N*/(*d*_60_ − *d*_45_), (6)

where *N* is the rolling speed of the rubber tire and *N* is generally set as 42 cycle/min, *d*_45_ and *d*_60_ are the deflections at 45 min and 60 min, respectively.

#### 2.4.3. Low-Temperature Indirect Tensile Stiffness Modulus Test

**Figure 5:**
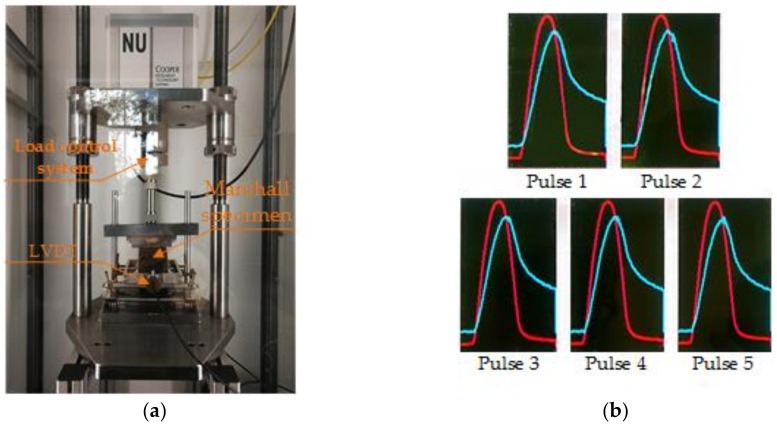
Indirect tensile stiffness modulus test: (**a**) indirect tensile stiffness modulus (ITSM) test; and (**b**) schematic diagram of the load.

The low-temperature tensile property is generally considered as an indicator for evaluating the anti-cracking ability and the indirect tensile stiffness modulus (ITSM) test (Cooper Research Technology Ltd., Ripley, UK) was adopted and conducted, according to the standard AASHTO TP-31, which is shown in Figure 5a [34]. A universal testing machine was used to perform the ITSM test. First, the Marshall specimens were put in an environment at 5 and 20 °C, for at least 5 h. Second, three replicate specimens were measured for ITSM and the load shown in Figure 5b was applied. The detailed parameters of the load can be found in a previous study [34]. Then the indirect tensile stiffness modulus (*S_m_*) could be obtained by calculation, as follows:

*S_m_* = *F* × (*μ* + 0.27)/(*h* × *Z*)*,* (7)

where *F* is the maximum loading (N); *μ* is the Poisson ratio, and *μ* = 0.25 and 0.35, at 5 and 20 °C; *h* is the specimen height (mm); *Z* is the horizontal deformation (mm).

#### 2.4.4. Moisture Stability Test

**Figure 6:**
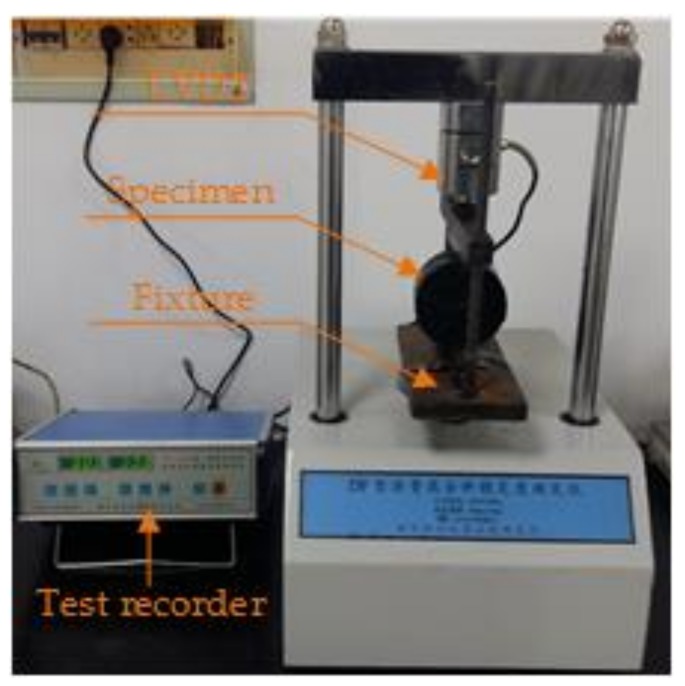
The freeze-thaw splitting test in this paper.

Freeze-thaw splitting test (shown in Figure 6, Nanjing Tuoxing Instrument Institute, Nanjing, China) is considered to be effective for analyzing the moisture stability of asphalt mixture and it has been widely used in many studies [22,23,24]. In accordance with rule T0729 in the JTG E20-2011 [30], the freeze-thaw splitting test was carried out at 25 °C, by the following steps:

(i) Marshall specimens were prepared and then divided into two groups, namely, the test group and the control group.
(ii) The test group was pretreated in a special condition, first, placed in water by vacuum saturation, after that put in the normal pressure condition.
(iii) Subsequently, the pretreated test group was conditioned at a low temperature of −18 °C for about 16 h, after that were placed in water at a temperature of 60 °C, for one day.
(iv) Both, the test and the control groups were immersed into water of 25 °C, for at least 2 h.
(v) The Marshall specimen was placed, centrally, in the Marshall apparatus and a loading force with a speed of 50 mm/min was loaded onto the specimen, until the specimen was broken.

The splitting tensile strength could be calculated by Equations (8) and (9):

*R*_T1_ = 0.006287 × *P*_T1_/*h*_1_, (8)

*R*_T2_*=* 0.006287 × *P*_T2_/*h*_2_, (9)

where *R*_T1_ and *R*_T2_ are the control group and the test group, respectively; *P*_T1_ and *P*_T2_ are the maximum loads of the control and the test groups; *h*_1_ and *h*_2_ are the heights of the control and the test groups. Furthermore, the freeze-thaw splitting tensile strength ratio (*TSR*) could be obtained as follows:

(10) $$TSR=({\bar{R}}_{T2}/{\bar{R}}_{T1})\;\times \;100,$$

where ${\bar{R}}_{T1}$ and ${\bar{R}}_{T2}$ are the control group and the test groups, respectively.

## 3. Results and Discussion

### 3.1. Determination of the Optimum Asphalt Content Using the Marshall Design Method

**Figure 7:**
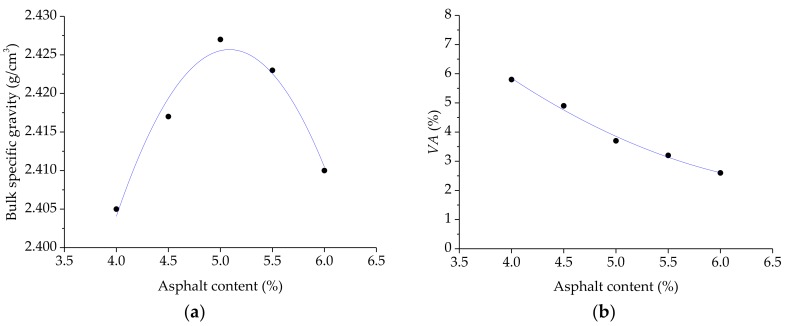

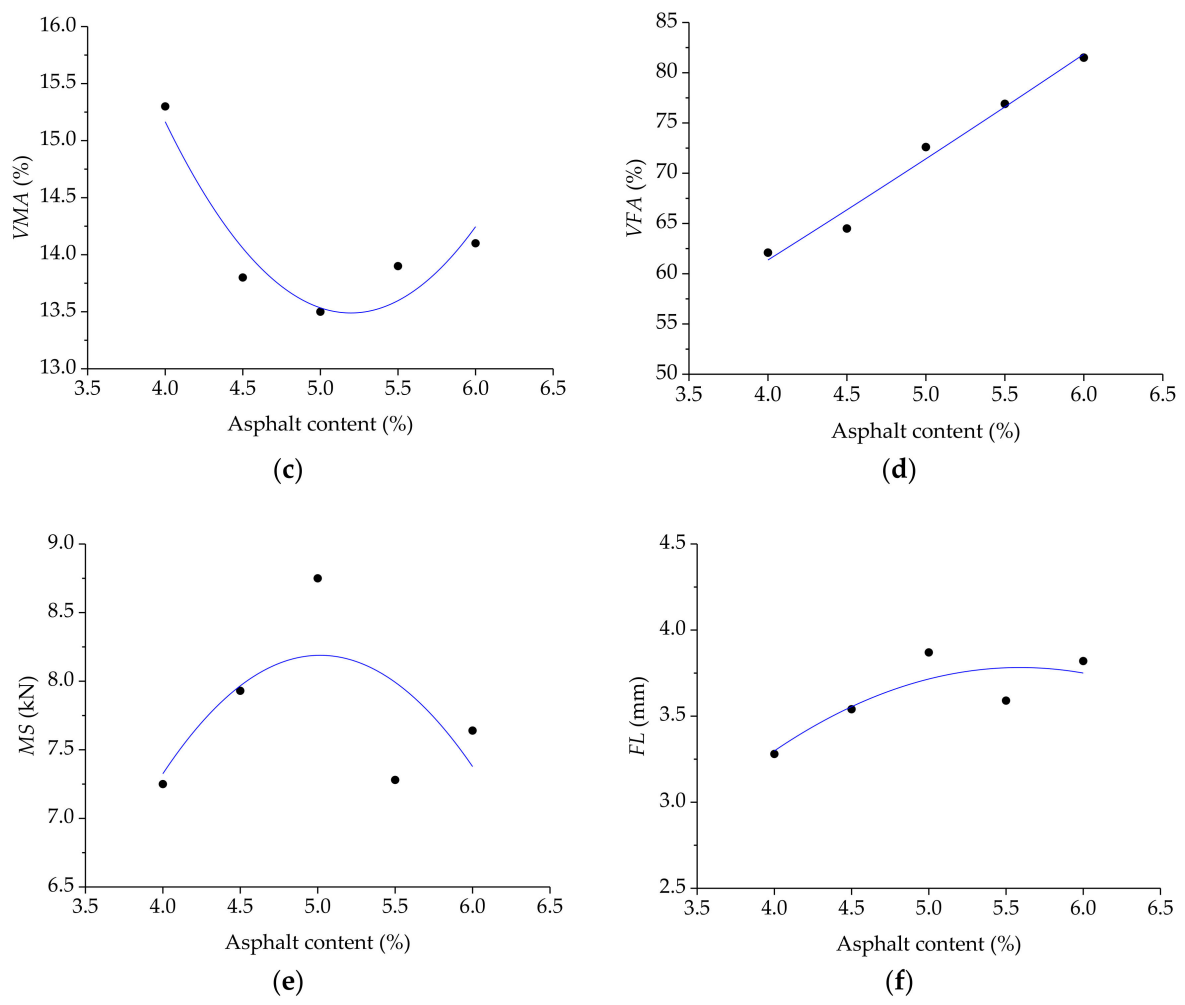
The Marshall design results of asphalt mixture without basalt fiber: (**a**) Bulk specific gravity; (**b**) *VA*; (**c**) *VMA*; (**d**) *VFA*; (**e**) *MS*; and (**f**) *FL*.

Asphalt mixtures with different basalt fiber proportions of 0% (control), 0.2%, 0.3%, 0.4%, and 0.5% by mass, were prepared, and were denoted by group 1 (control), 2, 3, 4, and 5, respectively. Then, the optimum asphalt content for these asphalt mixtures needed to be obtained, using the Marshall design method [29,35]. For each group, a range of the asphalt-aggregate ratios from 4.0% to 6.0% with an increment of 0.5%, was designed and tested by the Marshall design method. Figure 7 shows the Marshall design results of the asphalt mixtures (control group 1), including bulk specific gravity, *VA*, *VMA*, *VFA*, as well as *MS* and *FV*. Therefore, the optimum asphalt content (OAC) of the asphalt mixture, without the basalt fiber (control group 1) could be determined through the maximum density, maximum Marshall stability, and target air voids, and the OAC value was 5.03%. Afterwards, the OAC values of the other four groups of asphalt mixtures (i.e., group 2, 3, 4 and 5) could also be obtained and the OAC results are listed in Table 5. From Table 5, it could be seen that the OAC of different asphalt mixtures gradually increased with the basalt fiber content. This trend agrees with the results obtained in previous research, which may be attributed to the fact that basalt fiber has a larger specific surface area and the fibers can also absorb the light components in bitumen [9,36].

### 3.2. Optimum Basalt Fiber Content Based on the Pavement Performances

#### 3.2.1. High-Temperature Rutting Resistance

**Figure 8:**
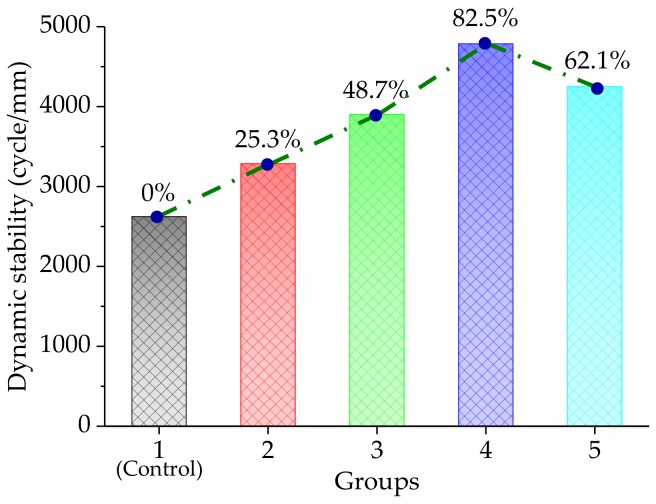
High-temperature rutting test results.

The rutting test was conducted at 60 °C for the asphalt mixtures with different basalt fiber contents, at the corresponding OAC values. Figure 8 shows the high-temperature rutting test results of the five groups. It could be clearly seen that the dynamic stability results demonstrated a rising trend, first, and then came down, when the basalt fiber content was increased, gradually. Furthermore, the dynamic stability reached the largest value, at a basalt fiber content of 0.4%. Compared to the control group, the dynamic stability results of the test groups were improved by, approximately, 25.3%, 48.7%, 82.5%, and 62.1%, respectively. Ordinarily, a larger *DS* value means a preferable anti-rutting [18]. Accordingly, the basalt fiber was proved to be able to well improve the rutting resistance of the asphalt mixture. This is because the basalt fiber was uniformly dispersed in the asphalt mixture and there was a spatial networking structure. Meanwhile, the basalt fiber could absorb some light components of bitumen to improve its viscosity [13]. Thus, the stability of the asphalt mixture can be reinforced by the addition of basalt fiber. However, it should be noted that the reinforcement of the basalt fiber slightly decreased. This may be attributed to the coagulated basalt fiber or the uneven dispersion of the basalt fiber in the bitumen, leading to weak points.

In addition, one-way analysis of variance (ANOVA) results using the Statistical Product and Service Solutions (SPSS) software (24.0, International Business Machines Corporation, New York, NY, USA) for high-temperature rutting test, are listed in Table 6. Tukey’s HSD (honest significant difference) test was used to perform the post hoc multiple comparisons and the results are listed in Table 7. From Table 6 and Table 7, the F-value was larger than F_0.01_(4,10) = 5.99, indicating that the basalt fiber content had a significant influence on the high-temperature property of the asphalt mixture, which was also proved by Tukey’s HSD results.

#### 3.2.2. Low-Temperature Indirect Tensile Stiffness Modulus

**Figure 9:**
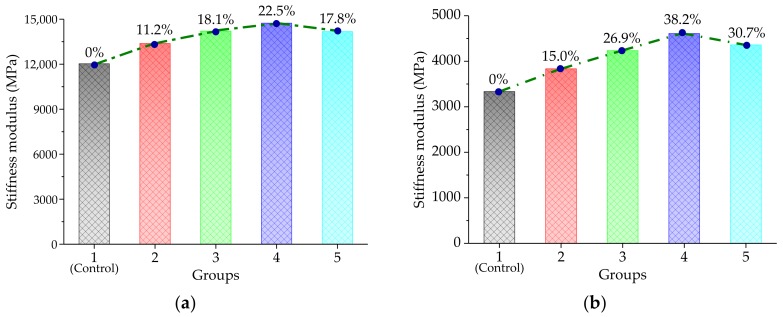
Low-temperature indirect tensile stiffness modulus test results: (**a**) At 5 °C; and (**b**) at 20 °C.

An indirect tensile stiffness modulus (ITSM) test was conducted at 5 and 20 °C, to investigate the low-temperature properties of the asphalt mixture with basalt fiber. The indirect tensile stiffness modulus could be calculated by Equation (7), based on the test data. Subsequently, the low- temperature indirect tensile stiffness modulus test results are plotted in Figure 9.

As shown in Figure 9, it could be observed that when the basalt fiber content increased continuously, the ITSM values also presented the variation trend of first increasing and then decreasing. Generally, the indirect tensile stiffness modulus is an indicator to evaluate the low- temperature anti-cracking ability, and a larger ITSM value of the asphalt pavement stands for a better anti-cracking ability. As shown in Figure 9, the low-temperature anti-cracking performance was improved with the addition of basalt fiber. With respect to the control group, the ITSM values increased by 11.2%, 18.1%, 22.5%, and 17.8% at 5 °C and 15.0%, 26.9%, 38.2%, and 30.7% at 20 °C, when adding basalt fiber of 0.2%, 0.3%, 0.4%, and 0.5% concentrations. In addition, it was evident that the ITSM had the most significant effect when the basalt fiber content was 0.4%. This variation trend might have been caused by the spatial networking structure of the basalt fiber, in the asphalt mixture. The absorption between bitumen and basalt fiber lead to a higher proportion of structural bitumen, improving the interfacial bond strength. Meanwhile, the addition of the basalt fiber could also prevent a further expansion of the cracks. The decreasing ITSM may be also attributed to the uneven dispersion of basalt fiber in bitumen.

In addition, one-way analysis of variance (ANOVA) results, using the SPSS software for low-temperature ITSM test are listed in Table 8. Tukey’s HSD (honest significant difference) test was used to perform post hoc multiple comparisons and Tukey’s HSD results are listed in Table 9. From Table 8 and Table 9, the F-value was larger than the F_0.01_(4,10) = 5.99, indicating that the basalt fiber content had a significant influence on the low-temperature property of the asphalt mixture, which was also proved by Tukey’s HSD results.

#### 3.2.3. Moisture Stability Properties

**Figure 10:**
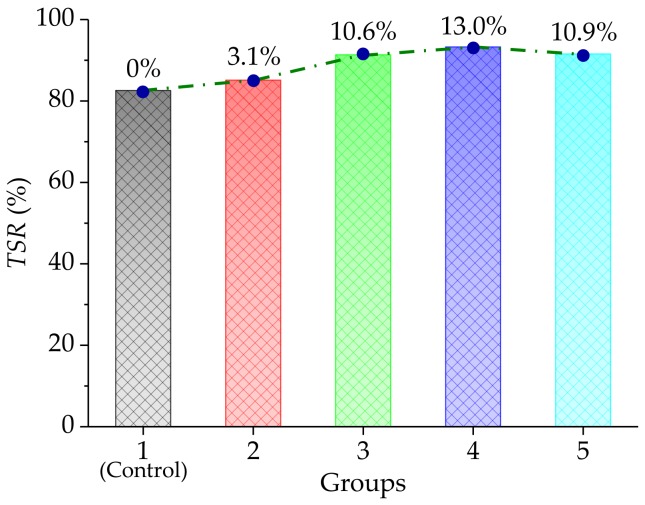
Moisture stability results of the freeze-thaw splitting test.

The freeze-thaw splitting test was carried out at test temperature of 25 °C, so as to explore the effect of basalt fiber on the moisture stability of the asphalt mixture. The freeze-thaw splitting tensile strength ratio (*TSR*) was used as an indicator calculated by the Equation 10 and the test results are illustrated in Figure 10.

In Figure 10, the *TSR* values exhibited, approximately, similar variation trends to the *DS* and the *ITSM*. The *TSR* values of test groups were improved by 3.1%, 10.6%, 13.0%, and 10.9%, compared with the control group, and the test group 4 with a basalt fiber content of 0.4% had the highest *TSR* value. This was expected, due to the absorption effect between the bitumen and the basalt fiber, the adhesion capability between the bitumen and the aggregates were improved, significantly, so that there was a difficulty in the exfoliation of the aggregates, under the effect of water. Simultaneously, basalt fiber with a high modulus and strength formed a spatial networking structure in the asphalt mixture, playing the role of reinforcement and toughening.

In addition, one-way analysis of variance (ANOVA) results, using the SPSS software for moisture stability tests are listed in Table 10. Tukey’s HSD (honest significant difference) test was used to perform post hoc multiple comparisons and Tukey’s HSD results are listed in Table 11. From Table 10 and Table 11, the F-value was larger than the F_0.01_(4,10) = 5.99, indicating that the basalt fiber content had a significant influence on the moisture stability of the asphalt mixture, which was also proved by Tukey’s HSD results.

In view of the pavement performances of the control and the test groups, when the basalt fiber increased, the rutting resistance, the anti-cracking and the moisture stability of the asphalt mixture were first improved and then slightly decreased, in which the pavement performances were improved, significantly, by adding basalt fiber of about 0.4%. Therefore, excessive basalt fiber content is not recommended and the optimal basalt fiber content was chosen as 0.4% and the corresponding optimum asphalt content was set as 5.35, in this study. The selected basalt fiber content and asphalt content were used to further investigate the water-temperature influences on asphalt mixtures.

### 3.3. Comparative Analysis of Damage Characteristics of the Asphalt Mixture under the Freeze-thaw Cycles

Asphalt mixtures modified by basalt fiber content of 0.4% and asphalt mixtures without basalt fiber, were prepared at the corresponding optimum asphalt content, and were divided into the test group and the control group, respectively. Before the test, the control and the test groups were immersed into water and under vacuum (98.0 kPa), for 15min, and soaked under atmospheric pressure, for 30 min. Then, the freeze-thaw cycles were carried out on both groups, in which the freezing condition was set as −18 °C, for 16 h, and the thaw condition was in water, at 60 °C for 8 h. After 0, 1, 3, 6, 9, 12, and 15 freeze-thaw cycle air voids, the splitting test, at 15 °C, and the ITSM test, at 10 °C, were carried out for further comparative analysis.

#### 3.3.1. Analysis of Air Voids

**Figure 11:**
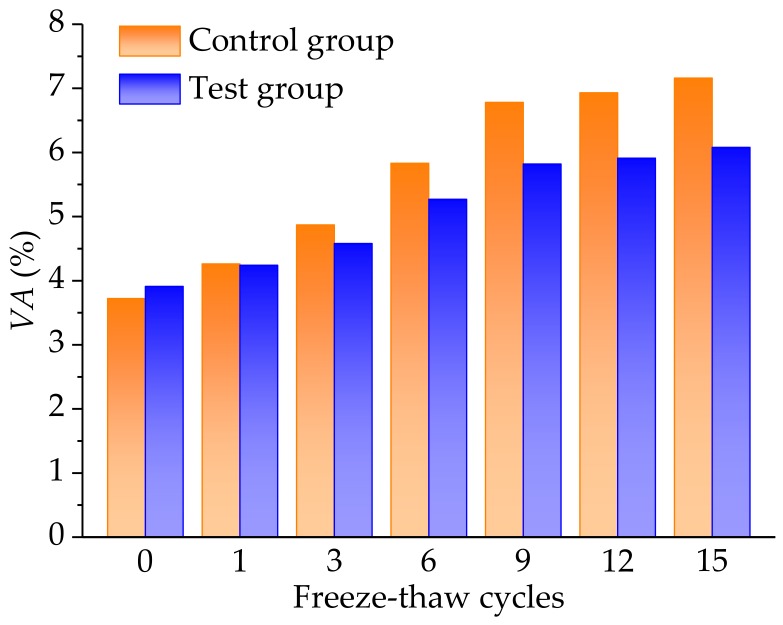
Comparative results of air voids of the control and the test groups, under different freeze-thaw cycles.

Air voids of the control and the test groups were measured and could be calculated by CT and DIP technologies. The CT, DIP technologies, and statistical methods were adopted for the control and the test groups, before and after the freeze-thaw cycles. The process could include the following steps: (1) CT image scanning; (2) image enhancement; (3) image denoising; (4) threshold cutting and binarization of images; and (5) air voids calculation. Figure 11 shows the air voids results of both the control and the test groups, under different freeze-thaw cycles.

As shown in Figure 11, it can be clearly observed that the air voids results of asphalt mixtures gradually increased as the freeze-thaw cycles increased. Furthermore, the rising trend of air voids of the asphalt mixtures was significant but the variation presented a slow trend when the freeze-thaw cycles exceeded 9 cycles. It is worth noting that the initial air voids of the control group, without the basalt fiber, were slightly lower than that of test group with basalt fiber. This was because it was relatively difficult to compact the asphalt mixtures with basalt fiber, due to the higher elastic modulus and reinforcement effect of basalt fiber. Under the action of the freeze-thaw cycles, the internal structure of the asphalt mixtures was damaged due to the volume expansion and temperature stress. Before the nine freeze-thaw cycles, the air voids first extended and then the adjacent air voids were coalesced in the asphalt mixture, leading to the significant variation trend, however, the expansion and formation of air voids became slow after the nine freeze-thaw cycles. In addition, the air voids of the test group were significantly lower than that of the control group. It was also evident that the basalt fiber formed a spatial networking structure, playing the role of reinforcement and toughening.

#### 3.3.2. Analysis of Splitting Strength

**Figure 12:**
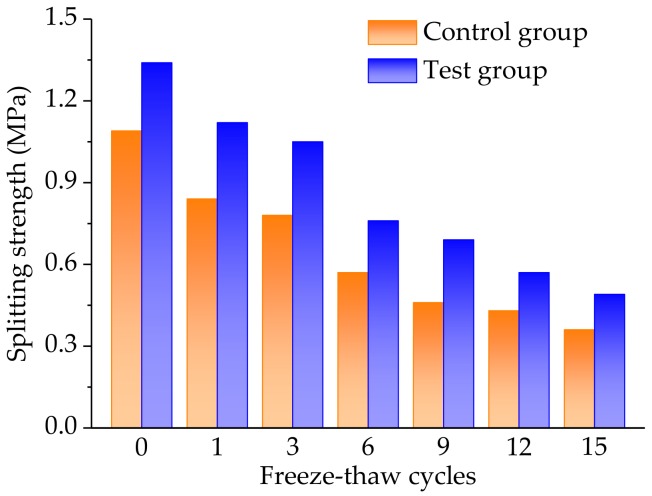
Comparative results of the splitting strength of the control and the test groups, under different freeze-thaw cycles.

Splitting strength of the control and the test groups could be obtained by the Equation 3, according to rule T0716 of the JTG E20-2011 [30]. Figure 12 illustrates the splitting strength results of both the control and the test groups, under the various freeze-thaw cycles.

As illustrated in Figure 12, the splitting strength values presented a decreasing trend with the freeze-thaw cycles and the splitting strength gradually decreased, slowly. Accordingly, the freeze-thaw cycle had a great effect on the mechanical properties of the asphalt mixture. This was because the adhesion capability between the bitumen and the aggregates became weaker and weaker, under the continuous action of the freeze-thaw cycles, resulting in a damaged internal structure of the asphalt mixture. Moreover, by a comparative analysis of the control and the test groups, the strength values of the test group were higher than those of the control group, under the same freeze-thaw cycles.

#### 3.3.3. Analysis of Indirect Tensile Stiffness Modulus

**Figure 13:**
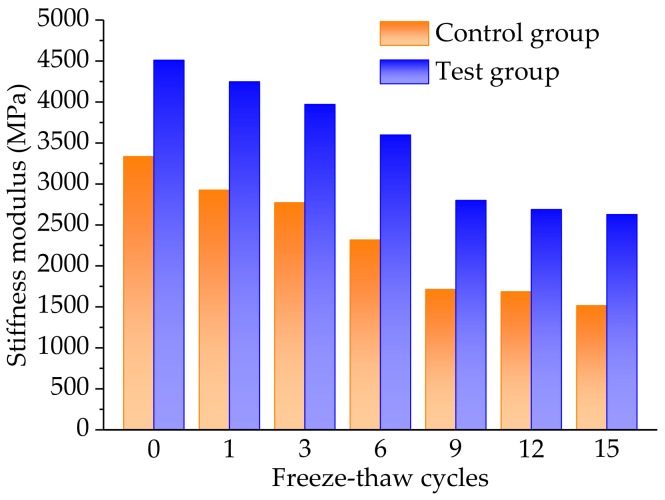
Comparative results of the indirect tensile stiffness modulus of the control and the test groups, under different freeze-thaw cycles.

The indirect tensile stiffness modulus of the control and the test groups could be calculated by the Equation 7 and the experimental procedure referred to in Section 2.4.3. Figure 13 plots the indirect tensile stiffness modulus results of both the control and the test groups, under different freeze-thaw cycles.

As plotted in Figure 13, the indirect tensile stiffness modulus results presented an, approximately similar, decreasing variation trend to the splitting strength in Figure 12. It was expected that indirect tensile stiffness modulus is also considered to be an indicator of the mechanical performance of the asphalt mixture and is connected with the bearing capacity of traffic loads. The freeze-thaw cycles had a negative effect on the mechanical properties of the asphalt mixtures. Meanwhile, the addition of basalt fiber into the asphalt mixture could significantly enhance the anti-cracking and mechanical properties of the asphalt mixtures, leading to a reinforcement mechanism.

## 4. Conclusions

The primary objective of this paper was to study the damage characteristics of asphalt mixtures (AC-13), reinforced with an eco-friendly basalt fiber, with a length of 6 mm, under the condition of freeze-thaw cycles. The optimum asphalt content and the optimum basalt fiber content were obtained by the Marshall design method and ordinary pavement performances. Then, the freeze-thaw cycle tests were performed for the control and the test groups of asphalt mixtures. The following conclusions could be drawn:

- The optimum asphalt content gradually increased with the addition of the basalt fiber content.
- Based on ordinary pavement performances, when adding basalt fiber, the pavement performances of the asphalt mixture exhibited a trend of first, increasing and then deceasing in performance. This was due to a spatial networking structure by the basalt fiber in the asphalt mixture. However, excessive basalt fiber was not good for asphalt mixture.
- Basalt fibers with higher content would be difficult to disperse, unevenly, in the bitumen, leading to weak points. Therefore, higher basalt fiber content is not recommended and the optimum basalt fiber content could be determined as 0.4% for further freeze-thaw cycle tests, according to the pavement performances of the asphalt mixtures.
- The freeze-thaw cycles had a negative effect on the mechanical properties of the asphalt mixtures. Adding basalt fibers into asphalt mixture could significantly improve the freeze-thaw resistance and the mechanical performance of the asphalt mixture, leading to a reinforcement mechanism.

## Figures and Tables

**Table 1:** Basic physical properties of the bitumen.

Properties		Measurement	Technical Criterion
Penetration @ 25 °C, 100 g, 5 s (0.1 mm)		88	80–100
Softening point (°C)		47	≥44
Ductility	@ 10 °C, 5 cm/min (cm)	43.5	≥30
	@ 15 °C, 5 cm/min (cm)	153	≥100
Flash point (°C)		318	≥245
Solubility (trichloroethylene, %)		99.8	≥99.5
Density @ 15 °C (g/cm^3^)		1.05	−
RTFOT			
Mass loss (%)		0.22	±0.8
Penetration ratio @ 25 °C (%)		66	≥57
Ductility	@ 10 °C, 5 cm/min (cm)	28	≥8
	@ 15 °C, 5 cm/min (cm)	89.3	≥20

**Table 2:** Physical properties of the aggregates.

Sieve Size (mm)	13.2	9.5	4.75	2.36	1.18	0.6	0.3	0.15	0.075
Apparent density (g/cm^3^)	2.803	2.781	2.774	2.760	2.713	2.720	2.699	2.647	2.700

**Table 3:** Physical properties of the limestone powder.

Properties	Apparent Density (g/cm^3^)	Hydrophilic Coefficient	Sieving Test	
			Size (mm)	Passing (%)
Values	2.728	0.76	0.6	100
			0.15	95.3
			0.075	82.5

**Table 4:** Physical properties of the basalt fibers.

Properties	Color	Length	Diameter	Specific Gravity	Tensile Strength	Elastic Modulus	Elongation at Break
Units	−	mm	*µ*m	g/cm^3^	MPa	GPa	%
Value	Golden brown	6	13	2.56	3200	>40	3.2

**Table 5:** Optimum asphalt content of asphalt mixtures with different basalt fiber contents.

Group	1 (Control)	2	3	4	5
Basalt Fiber Content (%)	0.0	0.2	0.3	0.4	0.5
Optimum Asphalt Content (%)	5.03	5.16	5.27	5.35	5.42

**Table 6:** One-way analysis of variance (ANOVA) for high-temperature rutting test.

Analysis	Sum of Squares	Degree of Freedom	Mean Square	F-Value	Significance
Between groups	8498025.6	4	2124506.4	836.8	**
Within groups	25388.0	10	2538.8		
Total	8523413.6				

Note: “**” is significant at the 0.01 level.

**Table 7:** Tukey’s honest significant difference (HSD) test results for high-temperature rutting test.

Within Groups	1 vs. 2	1 vs. 3	1 vs. 4	1 vs. 5	2 vs. 3	2 vs. 4	2 vs. 5	3 vs. 4	3 vs. 5	4 vs. 5
Mean difference	−663	−1278	−2164	−1628	−615	−1501	−965	−886	−350	536
Significance	**	**	**	**	**	**	**	**	**	**

Note: “**” is significant at the 0.01 level.

**Table 8:** One-way analysis of variance (ANOVA) for low-temperature ITSM.

Analysis	Sum of Squares	Degree of Freedom	Mean Square	F-Value	Significance
5 °C					
Between groups	13339074.0	4	3334768.5	4338.8	**
Within groups	7686.0	10	768.6		
Total	13346760.0				
20 °C					
Between groups	2992568.4	4	748142.1	613.6	**
Within groups	12192.0	10	1219.2		
Total	3004760.4				

Note: “**” is significant at the 0.01 level.

**Table 9:** Tukey’s HSD test results for low-temperature ITSM.

Within Groups	1 vs. 2	1 vs. 3	1 vs. 4	1 vs. 5	2 vs. 3	2 vs. 4	2 vs. 5	3 vs. 4	3 vs. 5	4 vs. 5
5 °C										
Mean difference	−1348	−2177	−2705	−2145	−829	−1357	−797	−528	32	560
Significance	**	**	**	**	**	**	**	**	**	**
20 °C										
Mean difference	−499	−898	−1275	−1024	−399	−776	−525	−377	−126	251
Significance	**	**	**	**	**	**	**	**	**	**

Note: “**” is significant at the 0.01 level.

**Table 10:** One-way analysis of variance (ANOVA) for moisture stability.

Analysis	Sum of Squares	Degree of Freedom	Mean Square	F-Value	Significance
Between groups	259.1	4	64.8	1134.6	**
Within groups	0.571	10	0.057		
Total	259.7				

Note: “**” is significant at the 0.01 level.

**Table 11:** Tukey’s HSD test results for moisture stability.

Within groups	1 vs. 2	1 vs. 3	1 vs. 4	1 vs. 5	2 vs. 3	2 vs. 4	2 vs. 5	3 vs. 4	3 vs. 5	4 vs. 5
Mean difference	−2.55	−8.77	−10.71	−8.97	−6.22	−8.16	−6.42	−1.94	−0.20	1.74
Significance	**	**	**	**	**	**	**	**	-	**

Note: “−” indicates insignificant correlation, “**” is significant at the 0.01 level.
